# Concordance between Wada, Transcranial Magnetic Stimulation, and Magnetoencephalography for Determining Hemispheric Dominance for Language: A Retrospective Study

**DOI:** 10.3390/brainsci14040336

**Published:** 2024-03-29

**Authors:** Negar Noorizadeh, Roozbeh Rezaie, Jackie A. Varner, James W. Wheless, Stephen P. Fulton, Basanagoud D. Mudigoudar, Leigh Nevill, Christen M. Holder, Shalini Narayana

**Affiliations:** 1 Division of Pediatric Neurology, Department of Pediatrics, University of Tennessee Health Science Center, Memphis, TN 38163, USA; nnooriza@uthsc.edu (N.N.); rrezaie@uthsc.edu (R.R.); jwheless@uthsc.edu (J.W.W.); sparkerfulton@gmail.com (S.P.F.); bmudigou@uthsc.edu (B.D.M.); cholder7@uthsc.edu (C.M.H.); 2 Neuroscience Institute, Le Bonheur Children’s Hospital, Memphis, TN 38103, USA; jackie.varner@lebonheur.org (J.A.V.); leigh.nevill@lebonheur.org (L.N.); 3 Department of Anatomy and Neurobiology, University of Tennessee Health Science Center, Memphis, TN 38163, USA

**Keywords:** intracarotid sodium amytal (Wada) testing, transcranial magnetic stimulation (TMS), magnetoencephalography (MEG), language mapping, hemispheric dominance, epilepsy, brain tumor

## Abstract

Determination of language hemispheric dominance (HD) in patients undergoing evaluation for epilepsy surgery has traditionally relied on the sodium amobarbital (Wada) test. The emergence of non-invasive methods for determining language laterality has increasingly shown to be a viable alternative. In this study, we assessed the efficacy of transcranial magnetic stimulation (TMS) and magnetoencephalography (MEG), compared to the Wada test, in determining language HD in a sample of 12 patients. TMS-induced speech errors were classified as speech arrest, semantic, or performance errors, and the HD was based on the total number of errors in each hemisphere with equal weighting of all errors (classic) and with a higher weighting of speech arrests and semantic errors (weighted). Using MEG, HD for language was based on the spatial extent of long-latency activity sources localized to receptive language regions. Based on the classic and weighted language laterality index (LI) in 12 patients, TMS was concordant with the Wada in 58.33% and 66.67% of patients, respectively. In eight patients, MEG language mapping was deemed conclusive, with a concordance rate of 75% with the Wada test. Our results indicate that TMS and MEG have moderate and strong agreement, respectively, with the Wada test, suggesting they could be used as non-invasive substitutes.

## 1. Introduction

Surgical resection emerges as one of the more effective treatment options for individuals with brain tumors or medically resistant epilepsy. The presurgical mapping of the language cortex before surgery plays a crucial role in surgical preparation, aiming to minimize potential postoperative deficits [1]. Although invasive, the intracarotid sodium amytal (Wada) test is considered the ‘gold standard’ for determining hemispheric dominance (HD), while cortical stimulation mapping (CSM) is the accepted benchmark for localizing specific language cortices [2,3]. Recognition of the limitations of these invasive language mapping methods has led to the development of safer, non-invasive alternatives, such as functional magnetic resonance imaging (fMRI) [4], magnetoencephalography (MEG) [5], and more recently, transcranial magnetic stimulation (TMS) [1]. Not only are these techniques non-invasive, but they also offer several advantages over CSM and Wada, including decreased morbidity and patient discomfort, absence of time constraints, readily performed in children, easily repeatable if needed, and cost-effectiveness [1,2,5,6].

Both fMRI and MEG language mapping protocols have been validated against invasive counterparts and are widely utilized in older children and adults [5,7,8,9]. However, younger patients and those with developmental delays, claustrophobia, or anxiety frequently struggle to comply with the test requirements, often requiring sedation and resulting in decreased success in about 30% of cases [10,11]. In contrast to fMRI and MEG, TMS can be applied to patients of all ages and cognitive abilities [1]. TMS shares sensitivity to direct neuronal signaling similar to MEG, but it differs from both MEG and fMRI as it involves brain stimulation rather than imaging of evoked neuromagnetic and hemodynamic activity. TMS functions through electromagnetic induction, where a current in a coil generates a magnetic field that passes through the scalp and skull, inducing current in the brain tissue below. Neurons depolarize and fire synchronously, causing excitatory or inhibitory effects depending on location and stimulation settings. Additionally, task performance during TMS can be closely monitored since it is carried out overtly, unlike MEG and fMRI, which require tasks to be performed covertly to prevent artifacts related to motion. TMS also allows for expressive language mapping in the clinical population needing sedation for MEG and fMRI. Therefore, TMS is especially promising for younger children or patients in whom other methods are not successful. These non-invasive methods (i.e., fMRI, MEG, and TMS) are approved by the US Food and Drug Administration (FDA) for presurgical mapping, and there is a growing emphasis on TMS and its potential to mitigate postoperative language deficits.

Just like CSM in language mapping, TMS delivers a series of pulses to the cortex, introducing random or out-of-phase currents to disrupt ongoing activity while the patient performs a task. When applied to language-specific areas, this process results in the creation of a ‘virtual lesion’ [12] and induces speech errors, thereby aiding in the identification of these cortices. Using TMS, language-specific regions in both hemispheres, including Broca’s area (Brodmann area [BA] 44 and 45), Wernicke’s area (BA 21 and 22), ventral premotor cortex (BA 6), auditory cortex (BA 41, and 42), parietal operculum (BA 43), supramarginal gyrus (BA 40), and angular gyrus (BA 39) have been identified. TMS, like the Wada test, surveys both hemispheres and, therefore, allows for the calculation of the laterality index (LI) based on the number and types of induced speech errors. Based on the LI value, the language-dominant hemisphere can be identified.

To date, the agreement between fMRI and MEG with the Wada test in determining the HD has been assessed [5,7,8,9,13], but the effectiveness of TMS compared to the Wada test remains unknown. Up till now, only one study has reported on the concordance of TMS with Wada in seven patients [14]. However, in this study, TMS application was limited to BA 44 and 45 in the frontal lobe and BA 22, 39, and 40 in the temporal lobe, and not all patients received stimulation in both hemispheres. Additionally, the calculation of the LI was not consistent with the accepted standards.

In this study, we conducted a retrospective comparison of our clinical language mapping techniques using TMS and Wada in patients undergoing brain surgery evaluation at our institution. The primary aim of this study was to examine the concordance between Wada and TMS in determining HD in individuals with epilepsy or brain tumors. Importantly, our study provides two unique contributions compared to previously published studies. Firstly, this is the first study to directly compare TMS and Wada in determining HD in a comprehensive manner, including mapping both hemispheres and the estimation of LI. Secondly, we evaluated a larger sample size of patients undergoing language mapping. As most of these patients also underwent MEG language mapping, we also investigated the concordance between MEG and Wada and thereby expanded on the previous reports of concordance between MEG and Wada [5].

## 2. Materials and Method

### 2.1. Patients

We conducted a retrospective chart review to identify patients who had both TMS and MEG and also underwent Wada language mapping as part of their presurgical evaluation between September 2010 and 2023 at Le Bonheur Children’s Hospital, Memphis, TN. Additionally, the majority of patients also underwent TMS motor mapping, continuous scalp video electroencephalogram monitoring, MEG interictal and somatosensory mapping, anatomical and functional MRI, and neuropsychological testing during their presurgical evaluation. This chart review was approved by the institutional boards at the University of Tennessee Health Science Center and Le Bonheur Children’s Hospital.

**Figure 1:**
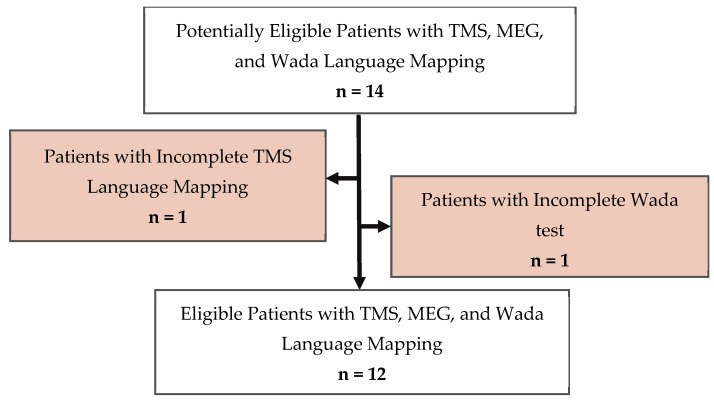
Standards for the reporting of diagnostic accuracy (STARD) flow chart studies of participants included in the study. We identified 12 patients with TMS, MEG, and Wada language mapping.

Of the 14 patients identified, 2 patients were excluded from the study (Figure 1). In one patient, only the left hemisphere was mapped with TMS, and the other patient was excluded because the Wada was not completed in the right hemisphere. Therefore, 12 patients between the ages of 11.78 and 25.16 were included in this study.

### 2.2. Anatomical MRI

Both TMS and MEG studies used each patient’s high-resolution T1-weighted brain MRI. MRI images were obtained on a GE Signa HDxt scanner (General Electric, Milwaukee, WI, USA). The T1-weighted 3D fast spoiled gradient-echo (FSPGR) sequence in the GE Signa HDxt scanner was acquired using an 8-channel head coil using the following parameters: 7.95/3.56 TR/TE, 12° flip angel, FOV = 512 × 512, and voxel size 0.5 × 0.5 × 0.8 mm.

### 2.3. Intracarotid Sodium Amytal (Wada) Testing

Our center adopted the Wada testing method described in previous publications [5,15,16]. The normal blood flow was confirmed in each patient by a cerebral angiogram, and no abnormalities affecting the Wada test interpretation were observed. Six patients showed some degree of cross-filling. A 5% sodium amobarbital solution was injected into the internal carotid artery via a catheter using a transfemoral approach. The initial bolus was administered manually over a 5-second interval, followed by additional infusions until contralateral hemiparesis was achieved. The hemisphere with the probable seizure focus was injected first, and the Wada test was then conducted. These procedures were repeated for the other hemisphere after an interval of at least 30 min. The mean dose of amobarbital for the anesthetizing left hemisphere was 111.5 mg (standard deviation (SD) = 16.4), while the mean for the right was 116.7 mg (SD = 18.0).

#### Wada Language Mapping

Prior to Wada testing, typically a day before, a baseline assessment encompassing activities such as picture naming, repetition, reading, and recognition memory was completed [15,16]. During the initial sodium amobarbital injection, patients were prompted to count forward to observe for speech arrest. Once contralateral hemiparesis was observed, the patient’s ability to follow simple instructions was assessed (e.g., sticking out the tongue), and a memory test was performed. Each item for the memory test was presented twice. Following the memory test, the following language measures were administered: (1) comprehension of one and two-step commands; (2) naming line-drawing objects; (3) sentence reading; and (4) repeating simple phrases. The protocol was then repeated in the other hemisphere after at least a 30 min rest period.

### 2.4. Transcranial Magnetic Stimulation (TMS)

We used an MRI-guided TMS system (Nexstim, Inc., Atlanta, GA, USA, with NexSpeech module) for language mapping. This system consists of a 3D tracking system, a TMS stimulator, and a simultaneous electromyography (EMG) acquisition system. The 3D tracking system includes an infrared camera (Polaris Vicra; Shelburne, VT, USA), a coil tracker, and a head tracker. A 70 mm figure-eight-shaped coil was used for stimulation. This coil has a maximum electric field strength of 172 V/m (at 25 mm from the coil surface), stimulating approximately 1 to 2 cm^2^ of the cortex beneath its central junction. Resting motor thresholds (rMT) were determined by recording electromyography (EMG) responses from hand muscles using self-adhesive disposable surface electrodes (Neuroline 720, Ambu Inc., Columbia, MA, USA). The rMT for the left hemisphere hand motor cortex was 50.17% ± 19.06% machine output (mean ± standard deviation), and for the right hemisphere hand motor cortex, it was 45.58% ± 8.11% machine output. The brain anatomy was linked to the locations of TMS stimulation (repetitive pulse protocol), and a cortical map representing the brain areas responsible for language functions was generated.

#### TMS Language Mapping Session

The TMS virtual lesion approach, as implemented in the NexSpeech module, was employed to identify the specific cortex responsible for language functions. Per our institution’s current individualized protocol, participants engaged in a task requiring them to name objects or colors. Object naming was the chosen task for all patients in this study. The participants were seated facing a monitor displaying the stimuli. Initially, their baseline performance (without TMS) was assessed by showing 50-line drawings of common objects for 1000 ms with interstimulus interval adjusted based on each participant’s response time (ranging from 3.5 to 5 s). They were asked to name the objects correctly and as quickly as possible. Stimuli erroneously named were discarded from the stimulus pool, ensuring that all stimuli presented during TMS had a corresponding correct baseline recording. Consequently, the final number of pictures used for each subject for mapping purposes differed, but it ranged between 20 and 50. Following baseline testing, patients repeated the naming task during TMS language mapping. TMS pulses at 5 Hz (five pulses) were administered in an event-related manner within a range of 0 to 300 ms relative to the onset of picture presentation [17] in order to target early language-related activity. The current flow of the coil was oriented anteroposteriorly to the temporal and frontal lobes. The stimulation intensity over the temporal and frontal lobes was adjusted between 35% and 45% machine output in order to produce an E-field of 80 to 120 V/m at a depth of 20 to 25 mm. In our cohort, this adjustment equaled 38.88% ± 4.15% (mean ± SD) and 36.75% ± 4.90% machine output for the temporal and frontal lobes, respectively. On average, the machine output used during temporal lobe mapping corresponded to 87% of rMT, and for frontal lobe mapping, the machine output was 82% of the rMT. While the patient was performing the task, TMS was administrated from the posterior aspect of the supramarginal gyrus and angular gyrus and moved anteriorly as much as the patient could tolerate. Following this, stimulation targeted the inferior and middle frontal gyri, which encompassed the pars opercularis and pars triangularis, alongside premotor and mouth motor regions. Although it has been previously demonstrated that a frequency of 5 Hz was effective and better tolerated by patients [18,19], any discomfort during TMS was monitored using a visual analog scale (VAS) for pain. This was assessed multiple times during mapping or when participants expressed discomfort [20]. If the pain level reached ≥3 on the VAS, the intensity was decreased but not below an E-field of 50 V/m. Mapping was stopped if the pain persisted or the E-field fell below 50 V/m. Each stimulus presentation, the participant’s response, and the cortical locations of TMS were recorded for subsequent analysis using NBS software 4.0.

### 2.5. Magnetoencephalography (MEG)

MEG language data were collected through a 4D MEG system (Magnes WH3600; 4-D Neuroimaging, San Diego, CA, USA) equipped with a total of 248 first-order magnetometer coils housed in a magnetically shielded room (MSR). Initially, a stylus was used to digitize the locations of five head position indicator (HPI) coils, which included fiducial points at nasion, left and right periauricular area, and two points on the forehead. The coils underwent brief activation by passing a small current through them, both at the beginning and end of the recording session. Their exact location in three-dimensional space was determined using a localization algorithm included in the recording system software. The patient’s head shape was also digitized using a stylus, and this digitization information, in conjunction with the localization of HPI coils referenced to the sensors, was employed to coregister the patient’s T1-weighted MRI image with the MEG data.

#### MEG Language Mapping Session

Receptive language mapping was carried out using a task modified from the continuous auditory word recognition paradigm [21]. First, the patient was placed in a supine position within the MSR, and a 5-meter-long plastic tube terminating in-ear inserts was placed into the patient’s outer ears. The auditory stimuli were presented individually for 1 s, with an interstimulus interval of 2–3 s, varied randomly. Just before the start of recording, patients were directed to listen to and lift their right index finger when they recognized one of the five target words (‘little’, ‘please’, ‘drink’, ‘jump’, and ‘good’). These target items were randomly interspersed among 40 other words in each of the three blocks, resulting in a total of 135 trials. All word stimuli were spoken by a native English speaker with a flat intonation and were digitally recorded at a sampling rate of 22,000 Hz and 16-bit resolution. Each patient underwent two consecutive sessions (Session 1 and Session 2) of language mapping.

### 2.6. Data Analysis

#### 2.6.1. Wada Data Analysis

A neuropsychologist scored the language tasks as normal, mildly, moderately, or severely deficient. A hemisphere was determined to have a critical language cortex if sodium amobarbital injection to that hemisphere disrupted language in at least two of the tests, with at least one task being moderately disrupted (or worse). A hemisphere was also determined to have a critical language cortex if three of the four tests were rated as mild disruption. If only one hemisphere met these criteria, a unilateral language representation was declared. If both or neither hemisphere met these criteria, language representation was deemed bilateral.

#### 2.6.2. TMS Data Analysis

**Figure 2:**
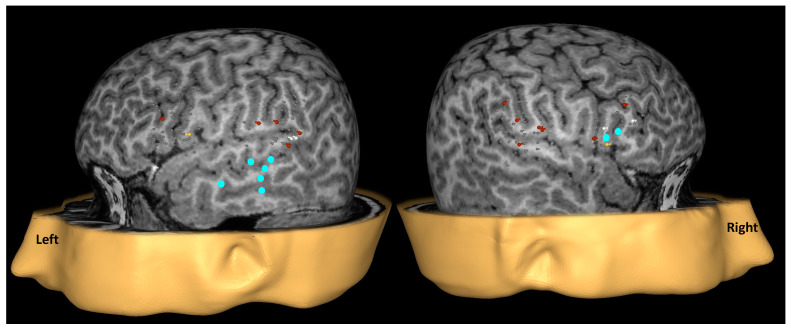
Example of language mapping with TMS and MEG in a 17-year-old right-handed male with a history of partial-onset seizures of left temporal lobe origin. Gray, white, yellow, and red pegs represent no error, speech arrest, semantic, and performance errors induced during TMS stimulation. TMS revealed right hemispheric dominance (HD) with a classic laterality index (LI) of −0.11 and bilateral HD with a weighted LI of −0.07. In MEG, brain activity sources were modeled as single equivalent current dipoles (ECD). Cyan circles represent dipoles’ location corresponding to the receptive language area identified by MEG. Although activation in both hemispheres was noted, greater number of dipoles were observed on the left. This finding implied left HD for receptive language.

The video recordings of naming during baseline and TMS were reviewed, and the patients’ naming performance during TMS was compared with their corresponding baseline responses. Speech errors during TMS were identified and characterized as follows: speech arrest, indicating the inability to produce any response; semantic errors, involving the substitution of the target word with a semantically related or unrelated word; performance errors, encompassing form-based distortions, slurring, stuttering, or imprecise articulation; hesitations, denoting delayed responses. Speech errors related to pain, discomfort, or distraction, as well as muscle stimulation, were excluded from the analysis. The cortical locations of all stimulations and each type of speech error were recorded in the native MRI coordinate system. Similar to fMRI [22] and MEG [23], TMS-induced speech and language errors can also be utilized for calculating a laterality index (LI). The classic LI is comparable to those employed in fMRI and MEG studies. The classic LI was calculated as (E_left_ − E_right_)/(E_left_ + E_right_), where E_left_ and E_right_ represent the total number of errors in the left and right hemispheres, respectively. A weighted LI [23] was also calculated by assigning higher weights to speech arrests (3×) and semantic errors (2×) compared to performance errors. The range 0.1 ≤ LI < 1 and −1 ≤ LI < −0.1 indicates left and right hemisphere dominance, while −0.1 ≤ LI ≤ 0.1 suggests bilateral representation. In Figure 2, an example of cortical locations targeted by TMS stimulation and the corresponding patient responses is illustrated. Based on the classic LI, the patient was deemed to have right HD, whereas the weighted LI suggested a bilateral HD for speech and language.

#### 2.6.3. MEG Data Analysis

For each language mapping session, the recorded data were digitized at 508 Hz and underwent offline processing, including bandpass filtering between 0.1 and 20 Hz. The data were baseline-corrected (150 ms prestimulus onset) to eliminate DC drifts, and a noise reduction algorithm, which was included in the 4D Neuroimaging software, was applied. Subsequently, the filtered event-related field (ERF) epochs were time-domain averaged. After visually examining the auditory ERF in each session, the session demonstrating better quality was selected. Using the chosen session, brain activity sources were modeled as single equivalent current dipoles (ECD) and independently fitted at successive 2-millisecond intervals. To ensure the adequacy of source solutions, we considered a correlation coefficient of at least 0.9 between the observed and the ‘best’ predicted magnetic field distribution. Additionally, we limited the source localization to the ERF segment between 200 and 800 milliseconds after stimulus onset, ensuring they primarily represented language-related activity and not modality-specific sensory activation. The estimated dipole location corresponding to receptive language mapping was then projected onto the patient’s T1-weighted MRI image. The HD in MEG language was determined by comparing the number of acceptable dipoles localized in the left and right hemispheres and the spatial extent of activation critical to supporting receptive language function. In Figure 2, the concentration of evoked activity sources (solid cyan circles projected onto the patient’s MRI) showed engagement of both hemispheres with a preponderance of the left, suggestive of left HD for receptive language.

## 3. Results

### 3.1. Patients

The patients’ demographic, clinical, and language HD details determined through various methods are summarized in Table 1. The dataset comprised seven females (58.33%) and five males (41.67%), with an average age of 18.76 years (range: 11.78–25.16 years, median age: 18.05 years). Among the 12 patients, 10 (83.3%) were right-handed. The information regarding hand dominance was obtained from the patients’ medical records and was based on patient self-report. All individuals underwent evaluation for epilepsy except for one case involving a left posterior mesial temporal tumor. Among the cases, the seizure focus/tumor was in the left hemisphere for nine patients (75%), whereas in three patients (25%), the seizure focus was on the right hemisphere.

### 3.2. Concordance between Wada, TMS, and MEG Findings on Hemispheric Dominance (HD)

Wada identified language HD to be left, right, and bilateral in seven, one, and four patients, respectively. Language HD was determined by TMS using the classic- and weighted LI, which was mostly consistent within each patient, except for a single case where the classic LI indicated right dominance, while the weighted LI indicated bilateral dominance. In the remaining patients, HD determined by TMS using two LI methods showed left, right, and bilateral dominance in eight, two, and one patient, respectively. Among the 12 patients, conclusive MEG language mapping was achieved in eight cases, with HD deemed left in 7 patients and bilateral in 1 patient.

The relationship between identified language HD by TMS using Wada, both classic- and weighted LI, and MEG procedures are presented in Table 2. Our findings indicate that HD lateralization using the classic LI in TMS aligns with Wada in seven (58.33%) patients (sensitivity 68.75%, specificity 75%, Cohen’s kappa 0.40, *p* < 0.05). Moreover, HD lateralization using the weighted LI in TMS is concordant with Wada in eight (66.67%) patients (sensitivity 75%, specificity 75%, Cohen’s kappa 0.47, *p* < 0.05). In the case of eight patients with conclusive MEG language mapping, HD lateralization by MEG is consistent with Wada in six (75%) of patients (sensitivity 88.89%, specificity 85.72%, Cohen’s kappa 0.75, *p* < 0.05). Our study demonstrates that TMS exhibited moderate agreement with the Wada findings, while MEG showed strong agreement with Wada in determining HD.

### 3.3. Postoperative Results

Patients one, two, five, six, seven, nine, and twelve had lesions in the left temporal lobe, all of whom underwent left temporal lobectomy (Table 2). Among these, four out of seven patients did not experience any postoperative deficits. Post-surgery language assessments for patient one revealed permanent impairment in language task performance and word-finding difficulties. Patient two experienced mild aphasia following surgery, necessitating speech therapy to address persistent expressive communication difficulties. Patient twelve also encountered a transient language deficit that required speech therapy after the procedure. Patients three, four, and ten, whose seizures originated from the right temporal lobe, underwent right temporal lobectomy without encountering postoperative deficits. Similarly, patient eleven, diagnosed with a left posterior mesial temporal tumor, underwent tumor resection without experiencing any deficits. Patient eight, diagnosed with complex partial seizures originating from the left posterior temporal–parietal lobe, underwent left frontotemporal craniotomy for the placement of a responsive neurostimulation device (RNS) with no language deficit.

## 4. Discussion

Brain surgery serves as a treatment for individuals struggling with brain tumors or medically resistant epilepsy. It is imperative to identify critical language before undertaking surgery. However, the invasive nature and limitations of the Wada test prompt the exploration of suitable non-invasive alternatives. TMS and MEG emerge as such tools for language mapping. This study explores the concordance between language mapping using Wada, TMS, and MEG in individuals having epilepsy or brain tumors. Our findings reveal that the classic- and weighted LI in TMS align with Wada in 58% and 67% of patients, respectively. Among the eight patients with conclusive MEG language mapping, there was a 75% concordance between MEG and Wada.

In this study, we examined a cohort of patients with epilepsy or brain tumors, mostly originating from the left temporal lobe. Patients two, six, seven, and twelve, who underwent Wada, TMS, and MEG language mapping, had a left temporal lobe lesion. HD, as determined by all tests, was consistent, except for patient six, where MEG language mapping was inconclusive due to motion artifacts. Subsequently, all four patients underwent left temporal lobectomy. Patient two, who was deemed to be left HD, experienced mild aphasia post-surgery, with speech therapy addressing expressive communication difficulties. Similarly, patient twelve was also found to be left HD by all modalities that required speech therapy after the procedure. These findings could be explained as these two patients were found by TMS and MEG to be strongly left hemisphere dominant with little engagement of right hemisphere areas. Patient six was found to have bilateral representation for language by both Wada and TMS and therefore, it was expected that he would not have speech or language deficits post-surgery. In the case of patient 7, despite the seizure focus being on the left temporal lobe and all modalities indicating left hemisphere dominance, the language area was located posterior to the seizure focus (left mesiotemporal). As a result, language function remained intact following the surgery.

Patients one, five, and nine, also diagnosed with left temporal lobe epilepsy, presented challenges in HD determination. Patient one exhibited inconsistency between Wada (right HD), TMS (left HD), and MEG (bilateral HD). Following a left temporal lobectomy, post-surgery language assessments revealed impaired language task performance and word-finding difficulties persisting beyond two years. In this patient, TMS and MEG had identified critical language areas in the left hemisphere, contrary to the Wada. Patient five (see Figure 2) showed discordant results in HD determination between Wada (bilateral HD), TMS (right HD using classic LI), and MEG (left HD), while Wada and TMS using the weighted LI were in agreement. Post temporal lobectomy, he did not have any speech or language deficits supporting bilateral dominance, as suggested by Wada and TMS. For patient nine, Wada and MEG consistently determined left HD, while TMS indicated significant language areas in the right hemisphere. This patient also underwent a left temporal lobectomy without exhibiting any postoperative deficits, suggesting that language functions post-surgery were being supported by the right hemisphere.

Patients three, four, and ten, with seizures originating from the right temporal lobe, displayed left HD dominance in all diagnostic tests, except for patient four, where the MEG results were inconclusive due to motion artifacts. All three patients underwent right temporal lobectomy without experiencing postoperative deficits, thereby confirming their left HD. Patient eight, diagnosed with complex partial seizures from the left posterior temporal–parietal lobe, exhibited inconsistent HD results between Wada (bilateral HD) and TMS (left HD). The MEG results were inconclusive due to motion artifacts. This patient underwent subdural grid placement to confirm the seizure onset zone and localize the language cortex. Cortical stimulation mapping confirmed the presence of the language cortex in the left temporal lobe, also localized previously by TMS. The language cortex was found to be in close proximity to the seizure onset zone. The patient, therefore, underwent left frontotemporal craniotomy for the placement of a responsive neurostimulation device (RNS). Patient eleven, evaluated for a left posterior mesial temporal tumor, demonstrated disagreement between Wada (bilateral HD) and TMS (right HD) in HD determination. The MEG results were inconclusive due to excessive myogenic artifacts, and the patient underwent tumor resection without postoperative deficits. These findings once again provide credence that the speech and language areas identified in the right hemisphere by Wada and TMS likely play a role in preserving/supporting post-surgical language function.

To date, several studies have explored the concordance of Wada and MEG in determining HD in both adults and children [5,9,24]. In one particular study, a cohort of 100 patients (aged 8–56 years) with medically intractable seizure disorder underwent Wada and MEG testing to determine HD for language [5]. The MEG and Wada data exhibited a high degree of concordance, reaching 87%. HD, as defined by MEG, demonstrated an overall sensitivity of 98%, with a specificity of 83%. Although our study involved only 12 patients, and it is not possible to directly compare ours with one reported in [5], our results still demonstrate a strong agreement in determining HD between Wada and MEG.

There is only one study that explores the concordance of TMS with Wada in a limited sample of seven patients [14]. However, this study has several limitations. Firstly, the sample size was small, and some patients received TMS only in either Broca’s or Wernicke’s area. Furthermore, not all patients underwent stimulation in both hemispheres. In contrast, our study marks a significant advancement by providing the largest database to date, encompassing both hemispheres in all 12 patients, including Broca’s area (BA 44 and 45), Wernicke’s area (BA 21, and 22), ventral premotor cortex (BA 6), auditory cortex (BA 41, and 42), parietal operculum (BA 43), supramarginal gyrus (BA 40), and angular gyrus (BA 39). Additionally, the method used for calculating the laterality index in [14] is inconsistent with the published standard approach, thereby making a direct comparison impractical.

Overall, our results suggest that both TMS and MEG demonstrate commendable agreement with Wada, implying their potential as non-invasive substitutes. Notably, adjusting TMS error weighting enhanced the accuracy of calculating the laterality index, enabling a more precise determination of HD. The post-surgical language outcomes in the study also provide additional support for the validity of HD determined by TMS and MEG. Both the possibility of post-surgical deficits (as in patients 1, 2, 8, and 12) and the absence of deficits (as in patients 3, 4, 5, 6, 7, 9, 10, and 11) could be predicted prior to surgery non-invasively by TMS and/or MEG. Particularly, TMS identified language cortices in the non-lesioned hemisphere more frequently than Wada or MEG, a finding that suggested that in those patients, the language function would be preserved postoperatively. In this study, TMS and MEG provided additional information regarding the location of language-specific cortices, which was not available by Wada. While Wada is useful in broad lateralization, we recommend adding TMS and/or MEG to the presurgical evaluation as they can provide additional localization information.

## 5. Conclusions

In conclusion, our study addresses the need for precise language mapping in brain surgery for epilepsy or brain tumors. Given the limitations of the invasive Wada test, we explored non-invasive alternatives, TMS and MEG, and investigated their concordance with Wada. The results contribute to understanding hemispheric dominance concordance and represent a significant advancement in language mapping. This study is the first to evaluate our comprehensive TMS-derived hemispheric dominance against the Wada test. Although it only includes 12 patients, it is the largest series to date. The agreement between TMS and MEG with Wada suggests their potential as non-invasive tools, with TMS showing enhanced accuracy through adjusted error weighting in the laterality index calculation. Considering the risks associated with performing Wada [25] and reduced success in younger children (less than 50% for ages 4.7–10 years) [26], we demonstrate that TMS and MEG are safe, non-invasive, valuable alternatives for presurgical language mapping in patients with epilepsy or brain tumor. These findings open avenues for further refinement and exploration of non-invasive techniques in neurosurgical language mapping.

## Figures and Tables

**Table 1:** Demographic, clinical, and language hemispheric dominance for study cohort. The handedness was obtained from the patients’ medical records. ¥ shows patients evaluated for tumors.

Patient	Age (Years)	Gender	Handedness	Seizure/Tumor Focus	HD_Wada_	HD_C-TMS_	HD_W-TMS_	HD_MEG_	Deficit ND/TD/PD
1	11.78	F	L	LT	R	L	L	B	PD
2	16.68	F	R	LT	L	L	L	L	PD
3	21.55	M	R	RT	L	L	L	L	ND
4	21.40	F	R	RT	L	L	L	Inc	ND
5	17.29	M	R	LT	B	R	B	L	ND
6	14.40	M	R	LT	B	B	B	Inc	ND
7	25.05	F	R	LT	L	L	L	L	ND
8	25.16	F	R	LPT-P	B	L	L	Inc	ND
9	18.13	F	L	LT	L	R	R	L	ND
10	17.96	M	R	RT	L	L	L	L	ND
11 ^¥^	14.08	M	R	LPMT	B	R	R	Inc	ND
12	21.65	F	R	LT	L	L	L	L	TD

F (Female); M (Male); L (left); R (Right); B (Bilateral); Inc (Inconclusive); LT (Left Temporal); RT (Right Temporal); LPT-P (Left Posterior Temporal–Parietal); LPMT (Left Posterior Mesial Temporal); HD (Hemispheric Dominance); C-TMS (Classic Laterality Index for TMS); W-TMS (Weighted Laterality Index for TMS); ND (No Deficit); TD (Transient Deficit); PD (Permanent Deficit).

**Table 2:** Wada-based judgments on hemispheric dominance (HD) for language compared against HD determined by TMS using classic- and weighted laterality index (LI) and MEG procedures.

	HD_Wada_				HD_Wada_				HD_Wada_		
HD_C-TMS_	*L*	*B*	*R*	HD_W-TMS_	*L*	*B*	*R*	HD_MEG_	*L*	*B*	*R*
*L*	6	1	1	*L*	6	1	1	*L*	6	1	0
*B*	0	1	0	*B*	0	2	0	*B*	0	0	1
*R*	1	2	0	*R*	1	1	0	*R*	0	0	0

L (left); R (Right); B (Bilateral); HD (Hemispheric Dominance); C-TMS (Classic Laterality Index for TMS); W-TMS (Weighted Laterality Index for TMS).

## Data Availability

Data are unavailable since data sharing has not been approved by the Institutional Review Board.
